# Maternal 3,3-Dimethyl-1-Butanol Therapy Protects Adult Male Rat Offspring against Hypertension Programmed by Perinatal TCDD Exposure

**DOI:** 10.3390/nu13093041

**Published:** 2021-08-30

**Authors:** Chien-Ning Hsu, Chih-Yao Hou, Chien-Te Lee, Guo-Ping Chang-Chien, Sufan Lin, You-Lin Tain

**Affiliations:** 1 Department of Pharmacy, Kaohsiung Chang Gung Memorial Hospital, Kaohsiung 833, Taiwan; cnhsu@cgmh.org.tw; 2 School of Pharmacy, Kaohsiung Medical University, Kaohsiung 807, Taiwan; 3 Department of Seafood Science, National Kaohsiung University of Science and Technology, Kaohsiung 811, Taiwan; chihyaohou@webmail.nkmu.edu.tw; 4 Division of Nephrology, Kaohsiung Chang Gung Memorial Hospital, Kaohsiung 833, Taiwan; ctlee33@cgmh.org.tw; 5 Center for Environmental Toxin and Emerging-Contaminant Research, Cheng Shiu University, Kaohsiung 833, Taiwan; guoping@csu.edu.tw (G.-P.C.-C.); linsufan2003@csu.edu.tw (S.L.); 6 Super Micro Mass Research and Technology Center, Cheng Shiu University, Kaohsiung 833, Taiwan; 7 Department of Pediatrics, Kaohsiung Chang Gung Memorial Hospital and College of Medicine, Chang Gung University, Kaohsiung 833, Taiwan

**Keywords:** gut microbiota, aryl hydrocarbon receptor, 3,3-dimethyl-1-butanol, developmental origins of health and disease (DOHaD), hypertension, short chain fatty acid, trimethylamine-N-oxide, 2,3,7,8-tetrachlorodibenzo-p-dioxin (TCDD), renin-angiotensin system

## Abstract

Maternal exposure to environmental pollutants affects fetal development, which can result in hypertension in adulthood. Gut microbiota-derived metabolite trimethylamine (TMA), trimethylamine-N-oxide (TMAO), and short chain fatty acids (SCFAs) have been associated with hypertension. We tested a hypothesis that maternal 3,3-Dimethyl-1-butanol (DMB, a TMA inhibitor) therapy prevents 2,3,7,8-tetrachlorodibenzo-p-dioxin (TCDD) exposure-induced hypertension in adult offspring relevant to alterations of gut microbiota-derived metabolites, the mediation of aryl hydrocarbon receptor (AHR) signaling, and the renin-angiotensin system (RAS). Pregnant Sprague-Dawley rats were given weekly oral dose of TCDD 200 ng/kg for four doses (T), 1% DMB in drinking water (D), TCDD + DMB (TD), or vehicle (C) in pregnancy and lactation periods. Male progeny (*n* = 8/group) were sacrificed at the age of 12 weeks. Perinatal TCDD exposure caused hypertension in adult male offspring coinciding with reduced *α*-diversity, increased the *Firmicutes* to *Bacteroidetes* ratio, less abundant beneficial bacteria, impaired SCFA receptors’ expression, the activation of AHR signaling, and the aberrant activation of the RAS. Treatment with DMB during pregnancy and lactation rescued hypertension induced by perinatal TCDD exposure. This was accompanied by reshaping gut microbiota, mediating TMA-TMAO metabolic pathway, increasing acetic acid and its receptors, and restoring the AHR and RAS pathway. Our data provide new insights into the therapeutic potential of DMB, a microbiome-based metabolite treatment, for the prevention of hypertension of developmental origins.

## 1. Introduction

Hypertension is a common disease which can originate from early life [1]. An adverse environment in utero may induce morphological changes and functional adaption during kidney development, resulting in hypertension in later life [2]. This concept is referred to as “developmental origins of health and disease” (DOHaD) [3]. Maternal exposures to environmental pollutants can increase risk for developing many adult diseases, including hypertension [4]. A common environmental pollutant is 2,3,7,8-tetrachlorodibenzo-p-dioxin (TCDD). TCDD toxic effects are associated with the activation of the aryl hydrocarbon receptor (AHR) signaling pathway [5]. We and others have shown that maternal TCDD exposure increased the vulnerability of offspring for developing hypertension in adulthood [6,7,8].

Cumulative evidence suggests that gut microbiota and its metabolites contribute to the pathogenesis of hypertension [9,10,11]. Certain metabolites derived from gut microbes have been found to participate in the control of blood pressure (BP), such as trimethylamine N-oxide (TMAO) and short chain fatty acids (SCFAs) [12,13]. Dietary components like choline can be transformed to trimethylamine (TMA) by gut microbes. In the liver, TMA is subsequently oxidized by flavin containing monooxygenases (FMOs), to yield TMAO [14]. Exposure to dioxin-like pollutants has been associated with increased plasma TMAO levels [15]. However, whether maternal TCDD exposure can induce hypertension in adult progeny and whether it is associated with the mediation of the gut microbiota-dependent metabolic pathways remain to be elucidated. 

Since gut microbiota is highly relevant to hypertension, attention has been drawn to target on gut microbiota and related metabolites as new potential therapeutics to prevent hypertension of developmental origins [11]. Targeting TMA formation, 3,3-Dimethyl-1-butanol (DMB) can inhibit microbial choline TMA lyase activity to inhibit TMA and subsequent TMAO formation [16]. DMB occurs naturally in existing foods (e.g., extra virgin olive oils and grape seed oils) or alcoholic beverages (e.g., red wine). We previously found that DMB treatment in pregnancy and lactation protected adult offspring against maternal high-fructose diet-induced hypertension coinciding with alterations of the TMA-TMAO pathway and SCFAs [17]. Aberrant renin-angiotensin system (RAS) activation has emerged as another important mechanism behind the developmental programming of hypertension [18]. In another model of programmed hypertension, DMB therapy was reported to prevent adult offspring against hypertension coinciding with restoration of the balance of RAS and antagonization of AHR signaling [8]. These findings suggest that targeting on TMA-TMAO pathway by DMB might be a potential preventive therapy to act in several ways for the benefit against hypertension of developmental origins. Accordingly, we aimed to examine whether maternal DMB therapy prevents perinatal TCDD exposure-induced hypertension in adult progeny and explore the fundamental mechanisms.

## 2. Materials and Methods

### 2.1. Animal Study

**Figure 1:**
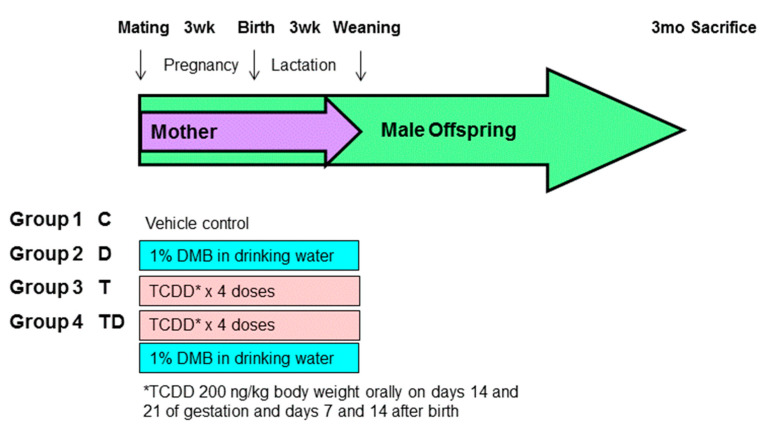
Experimental protocol used in the present study. *TCDD 200ng/kg body weight orally on days 14 and 21 of gestation and days 7 and 14 after birth.

Sprague–Dawley (SD) rats were purchased from BioLASCO Taiwan Co., Ltd., Taipei, Taiwan. The rats were housed in an AAALAC-accredited facility in our hospital. Food and water were available ad libitum. Individual female SD rat was placed with one male rat in a cage until mating was confirmed by the presence of a copulatory plug. As hypertension occurs at an earlier age and a higher rate in males than females [19], only male progeny was used in subsequent experiments. After birth, the subjects came from litters of eight pups to standardize the received quantity of milk and maternal pup care. Figure 1 illustrates the experimental protocol. Male offspring were allocated into four groups (*n* = 8 per group): control rats (C), rats treated with DMB (D), rats exposed to TCDD (T), and rats administered TCDD and DMB (TD). To construct a TCDD exposure model, pregnant dams received an oral dose of TCDD (Sigma-Aldrich, St. Louis, MO, USA) at 200 ng/kg body weight (BW) or corn oil vehicle (4 mL/kg BW) on gestational days 14 and 21 and days 7 and 14 after birth to cover the period of kidney development. The weekly dose of TCDD used here was based on prior research showing the half-life of TCDD in rats is approximately 3 weeks [8,20]. Half of the control or TCDD exposure pregnant rats received 1% DMB in drinking water in pregnancy and lactation. The dose was selected was based on prior work [16,17].

We used the CODA noninvasive BP system (Kent Scientific Corporation, Torrington, CT, USA) for determining the BP of 12-week-old offspring. To ensure accuracy and reproducibility, the rats were acclimated to restraint and tail-cuff inflation for one week prior to the experiment. The rats were placed on the specimen platform. Their tails were passed through tail cuffs and immobilized by adhesive tape. Following a 10-min warm-up period, 10 preliminary cycles of tail-cuff inflation were performed to allow the rats to adjust to the inflating cuff. A total of five cycles were recorded at each time point for each rat. Stable measures were taken and averaged. Fecal samples were collected (*n* = 8 per group) in the morning prior to sacrifice by lifting the tail and twisting it towards back to induce defecation. Later, collected fecal samples were frozen and placed into a −80 °C freezer. At 12 weeks of age, the rats were sacrificed with an i.p. overdose of pentobarbital (200 mg/kg). Heparinized blood samples were collected. The kidneys were subsequently collected. Plasma creatinine levels were analyzed by high performance liquid chromatography (HPLC) method, as we described previously [8].

Animal care and use was in strict accordance with the recommendations in the Guide for the Care and Use of Laboratory Animals of the National Institutes of Health. The experimental protocol was approved by the Institutional Animal Ethics Committee (IACUC) of Chang Gung Memorial Hospital (Permit Number 2017121408). 

### 2.2. Liquid Chromatography–Mass Spectrometry (LC–MS) Analysis

Plasma levels of TMAO, TMA, and their metabolites dimethylamine (DMA) were determined using a previously described method [8]. For the liquid chromatography–mass spectrometry (LC–MS) analysis, an Agilent 6410 Series Triple Quadrupole mass spectrometer (Agilent Technologies, Wilmington, DE, USA) with an electrospray ionization source was applied. We used diethylamine as an internal standard. Using an Agilent Technologies 1200 HPLC system, chromatographic separation was carried out on a SeQuant ZIC-HILIC column (150 × 2.1 mm, 5 μm; Merck KGaA, Darmstadt, Germany) protected by an Ascentis C18 column (2 cm × 4 mm, 5 μm; Merck KGaA). The eluate was monitored for DMA, TMAO, and TMA in multiple-reaction-monitoring mode using characteristic precursor-product ion transitions: *m*/*z* 46.1 → 30, *m*/*z* 76.1 → 58.1, and *m*/*z* 60.1 → 44.1, respectively.

### 2.3. Gas Chromatography-Mass Spectrometry (GC–MS) Analysis

Plasma concentrations of acetic acid, propionic acid, and butyric acid were determined by gas chromatography-mass spectrometry (7890B, Agilent Technologies Wilmington, DE, USA), as we previously published [8]. Analytical standard grades of acetic acid, propionic acid (Sigma-Aldrich, St. Louis, MO, USA), and butyric acid (Chem Service, West Chester, PA, USA) were used as standards. Chromatographic separation was carried out using a DB-FFAP column (30 cm × 0.25 mm, 0.25 µm; Agilent Technologies, Wilmington, DE, USA). We used 2-ethylbutiric acid as the internal standard. An injection volume of 1 μL was carried out at 240 °C, using a split ratio of 5:1. 

### 2.4. Quantitative Real-Time Polymerase Chain Reaction (qPCR) 

We determined the renal mRNA expression of SCFA receptors, components of the RAS, and AhR targeted gene by qPCR, following previously described methods [8]. RNA was extracted from each offspring’s kidney cortex and analyzed by qPCR. We used iCycler iQ Real-Time PCR Detection System (Bio-Rad, Hercules, CA, USA) and Quantitect SYBR Green PCR Reagents kit (Qiagen, Valencia, CA, USA) to perform two-step quantitative real-time PCR. We analyzed the following SCFA receptors: olfactory receptor 78 (Oflr78), G protein-coupled receptor 41 (GPR41), GPR43, and GPR109A. 

Additionally, we analyzed several RAS components, including angiotensinogen (AGT), renin, angiotensin converting enzyme (ACE), angiotensin converting enzyme-2 (ACE2), and angiotensin II type 1 and 2 receptor (AT1R and AT2R). Five AHR signaling pathway-related genes were determined, including AHR, aryl hydrocarbon receptor nuclear translocator (ARNT), aryl hydrocarbon receptor repressor (AHRR), TCDD-inducible poly-ADP-ribose polymerase (TIPARP), and cytochrome P450 CYP1A1 (CYP1A1). The R18S reference gene was used as the internal control as its constant expression across all samples. 

Table 1 provides the PCR primer sequences. All samples were assayed in duplicate. We calculated relative gene expression using the comparative threshold cycle (Ct) method. The fold-increase in the experimental sample, relative to the control, was calculated using formula 2^−ΔΔCt^.

### 2.5. Gut Microbiota Compositions

Stool samples were analyzed with metagenomics focused on the V3-V4 of the 16S DNA gene using the methods published previously [8]. The 16S rRNA amplicon sequencing libraries were prepared (Illumina, San Diego, CA, USA). We used the Illumina MiSeq platform sequencing (Illumina, San Diego, CA, USA) and analyzed next generation sequencing data using the Microbial Genomics Module of CLC Genomics Workbench 9.5.4 (Qiagen, Stockach, Germany). Illumina sequence data were carried out using QIIME version 1.9.1. The sequences were clustered into operational taxonomic units (OTUs) using the USEARCH algorithm with 97% sequence similarity threshold. Based on a representative sequence alignment with Fast-Tree, the phylogenetic relationships were constructed. We investigated the diversity patterns of microbial communities. Alpha diversity was measured by observing OTUs [21]. We accessed the β-diversity of gut microbiota using the Principal Coordinate Analysis (PCoA) across groups [22]. The linear discriminant analysis effect size (LEfSe) was sued to discover high-dimensional biomarkers. The threshold on logarithmic score (LDA) for discriminative features was set to 3.

### 2.6. Statistical Analysis

The Shapiro–Wilk normality test was used to determine if data were normal distributed. Data are expressed as mean ± SEM. Comparisons within three groups were analysis by one-way analysis of variance (ANOVA) followed by a Tukey’s post hoc test. A p-value less than 0.05 was considered statistically significant for all tests. We used the Statistical Package for the Social Sciences software (SPSS Inc., Chicago, IL, USA) to analyze all data.

## 3. Results

### 3.1. Morphological Values and Blood Pressures 

There was no death for all groups. The body weight (BW) was higher in the control compared to the other three groups (Table 2). Table 2 illustrates DMB caused a higher kidney weight vs. controls, whereas the kidney weight-to-BW ratio was not different among the four groups. Systolic BP (SBP) and mean arterial pressure (MAP) were highest in the T group. DMB therapy reduced diastolic BP in the TD group vs. the T group. Additionally, all four groups showed comparable levels of creatinine. 

### 3.2. TMA-TMAO Pathway

Table 3 illustrates that plasma concentrations of DMA and TMAO did not differ among the four groups. DMB therapy caused a higher plasma TMA level but a lower TMAO-to-TMA ratio in D and TD group compared to the controls. However, the plasma DMA-to-TMAO ratio was comparable among the four groups. 

### 3.3. Plasma SCFA Levels and Renal SCFA Receptors

We determined the most abundant SCFAs, acetic acid, propionic acid, and butyric acid in the plasma. As show in Table 4, plasma levels of acetic acid were higher in the D and T group than those in the controls. The increases of acetic acid were further augmented by the DMB treatment in the TD group. Additionally, plasma concentrations of propionic acid and butyric acid were no different among the four groups. 

**Figure 2:**
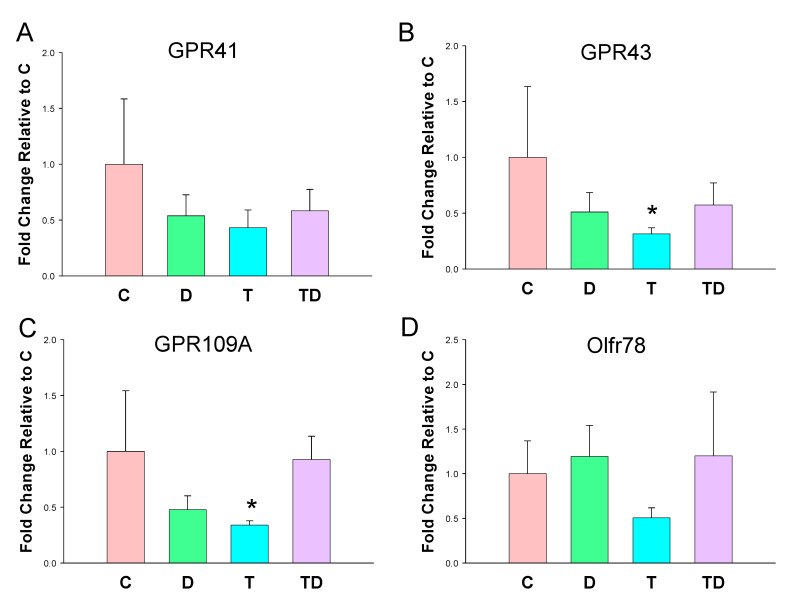
Effect of 3,3-Dimethyl-1-butanol (D) and 2,3,7,8-tetrachlorodibenzo-p-dioxin (T) on short chain fatty acid (SCFA) receptors. The mRNA expression of SCFA receptor (**A**) G protein-coupled receptor 41 (GPR41), (**B**) GPR43, (**C**) GPR109A, and (**D**) olfactory receptor 78 (Oflr78) in offspring kidneys. *N* = 8 per group; * *p* < 0.05 vs. C.

In view of SCFAs regulate BP via their receptors [23], we next analyzed mRNA expression of GPR41, GPR43, GRP109A, and Olfr78 in offspring kidneys. As shown in Figure 2, TCDD exposure had a tendency to reduce all SCFA receptor expression. However, statistical significance was only reached in GPR43 (Figure 2B) and GPR109A (Figure 2C). On the other hand, DMB had negligible effects on the renal mRNA expression of four SCFA receptors.

### 3.4. RAS and AHR Pathway

**Figure 3:**
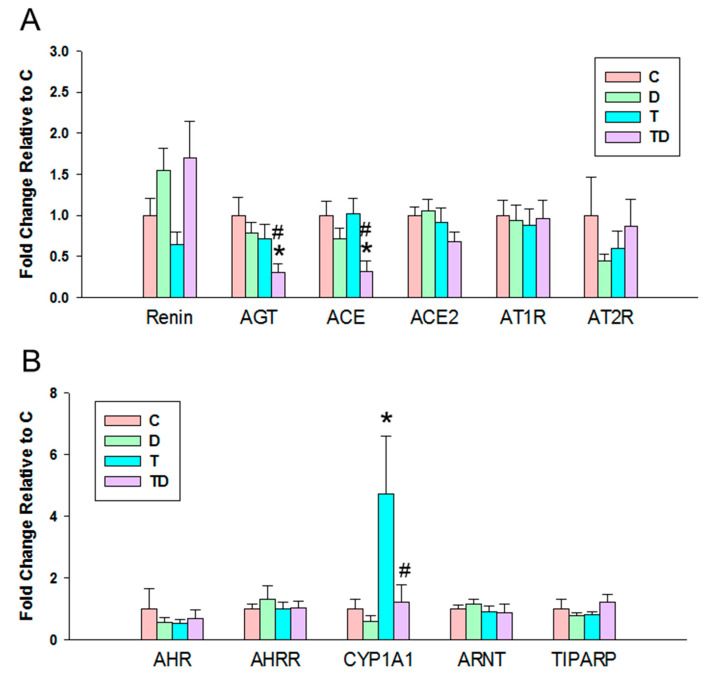
Effect of 3,3-Dimethyl-1-butanol (D) and 2,3,7,8-tetrachlorodibenzo-p-dioxin (T) on the mRNA expression of (**A**) renin-angiotensin system and (**B**) acryl hydrocarbon receptor (AHR) signaling pathway in offspring kidneys. AGT = angiotensinogen; ACE = angiotensin converting enzyme; ACE2 = angiotensin converting enzyme-2; AT1R = angiotensin II type 1 receptor; AT2R = angiotensin II type 2 receptor; AHR = aryl hydrocarbon receptor; AHRR = Aryl hydrocarbon receptor repressor; CYP1A1 = cytochrome P450 CYP 1A1; ARNT = Aryl hydrocarbon receptor nuclear translocator; TIPARP = TCDD-inducible poly-ADP-ribose polymerase; *N* = 8 per group; * *p* < 0.05 vs. C; ^#^ *p* < 0.05 vs. T.

In view of the dysregulation of the RAS and AHR pathways involved in programmed hypertension [18,24], we next evaluated the components of RAS and AHR target genes in offspring kidneys. We observed there was a significant decrease in mRNA expression of AGT and ACE in the TD group vs. the C and T group (Figure 3A), whereas there was no difference of mRNA expression of other components belonging to the RAS among the four groups.

Regarding AHR signaling pathway, Figure 3B illustrates that there was no difference in renal mRNA expression of AHR, AHRR, ARNT, and TIPARP. Renal mRNA expression of CYP1A1 was higher in the TCDD exposed-offspring, which was prevented by the DMB therapy in the TD group (Figure 3B). 

### 3.5. Gut Microbiota Compositions

**Figure 4:**
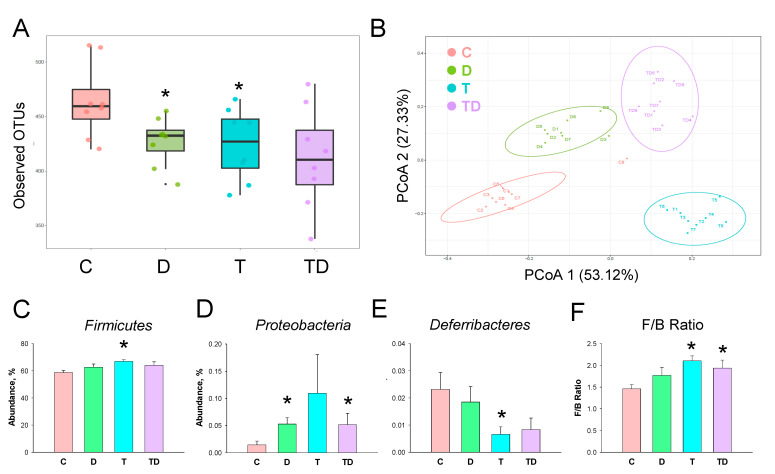
Effect of 3,3-Dimethyl-1-butanol (D) and 2,3,7,8-tetrachlorodibenzo-p-dioxin (T) on the gut microbiome in offspring. (**A**) α-diversity measured by the observed operational taxonomic units (OTUs). (**B**) β-diversity using the Principal Coordinate Analysis (PCoA). Relative abundance of the phylum (**C**) *Firmicutes*, (**D**) *Proteobacteria*, and (**E**) *Deferribacteres*. (**F**) The *Firmicutes* to *Bacteroidetes* (F/B) ratio. *N* = 8 per group; * *p* < 0.05 vs. C.

We next investigated how TCDD and DMB affected the gut microbiota compositions. Gut microbiome diversity was evaluated by measuring within and between communities (i.e., α- and β-diversity). Figure 4A illustrates that both D and T group had a lower α-diversity, represented as observed OTUs, compared to that in controls (both *p* < 0.05). We determined β-diversity by using PCoA plots to compare the bacterial community similarity. As shown in Figure 4B, scatterplots of PCoA analysis showed significant clustering according to study group, showing that the gut microbiota composition in the C group was distinctly reshaped by TCDD exposure, DMB administration, and their combined exposure. 

The phylum level abundance of *Firmicutes* was greater in the T group compared to the C group (Figure 4C). DMB treatment increased *Proteobacteria* abundance in the D and TD group vs. the C group (Figure 4D). TCDD exposure caused a reduction of phylum *Deferribacteres* abundance in the T group (Figure 4E). Additionally, the *Firmicutes* to *Bacteroidetes* (F/B) ratio, a microbial marker related to hypertension [25], was greater in the T and TD group than that in the C group (Figure 4F).

**Figure 5:**
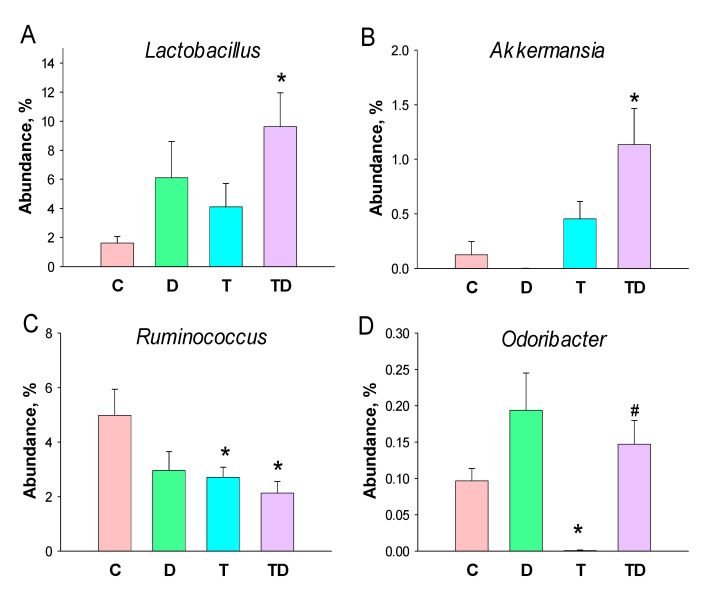
Effect of 3,3-Dimethyl-1-butanol (D) and 2,3,7,8-tetrachlorodibenzo-p-dioxin (T) on the gut microbiome in offspring. Relative abundance of the genera (**A**) *Lactobacillus*, (**B**) *Akkermansia*, (**C**) *Ruminococcus*, and (**D**) *Odoribacter*. *N* = 8 per group; * *p* < 0.05 vs. C; ^#^ *p* < 0.05 vs. T.

*Lactobacillus* and *Akkermansia* of the genus-levels were significantly higher in the TD group than those in the controls (Figure 5A,B). At the genus level, TCDD exposure caused a notable decrease in the abundance of *Ruminococcus* in the T and TD group (Figure 5C). Additionally, the abundance of genus *Odoribacter* was lower in the T group than that in the C group, which was restored by DMB treatment (Figure 5D).

**Figure 6:**
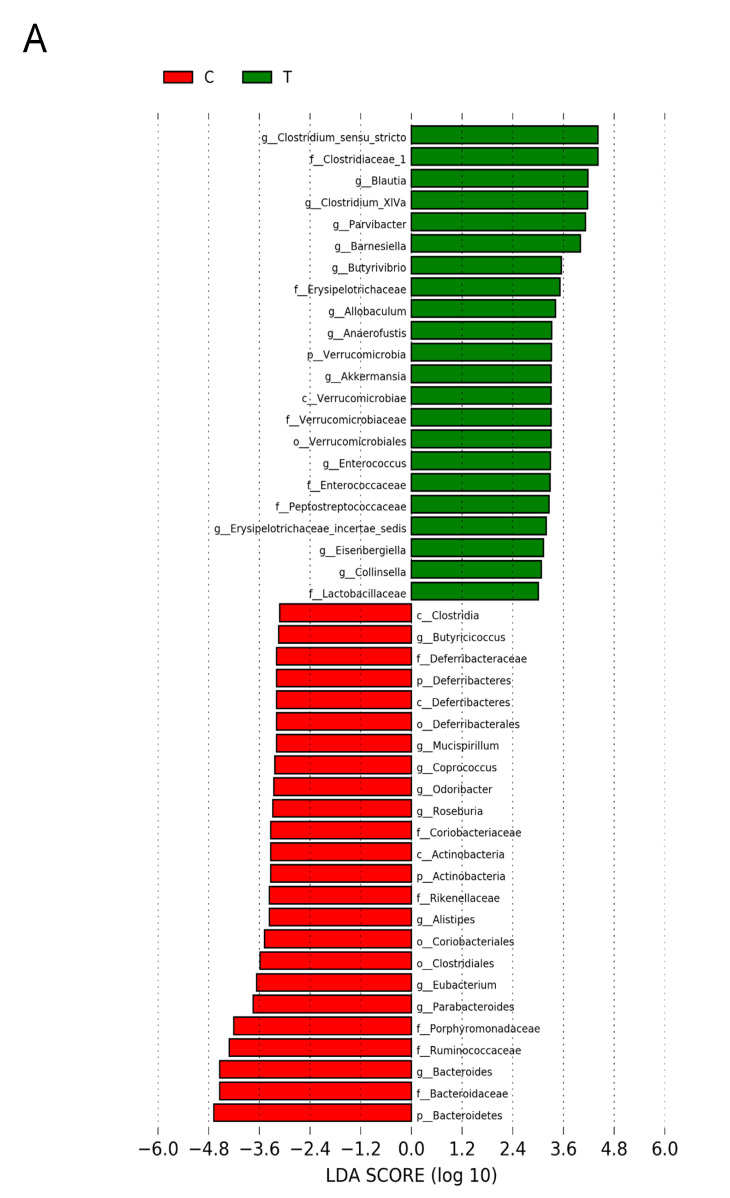

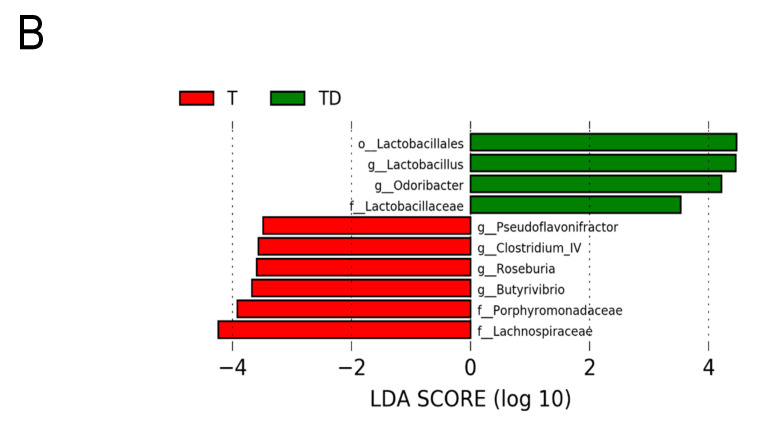
Effect of 3,3-Dimethyl-1-butanol (D) and 2,3,7,8-tetrachlorodibenzo-p-dioxin (T) on the gut microbiome in offspring. Linear discriminant analysis effect size (LEfSe) was carried out to identify microbial marker. Most enriched and depleted bacterial taxa in the (**A**) C (red) versus T group (green) and (**B**) T (red) versus TD group (green) are shown. The threshold on the linear discriminant was set to 3.

Figure 6 shows statistically significant microbial markers between groups, which were identified by the LEfSe analysis. There was a greater abundance of genera *Blautia, Akkermansia,* and *Collinsella*; whereas a lower abundance of *Roseburia, Alistipes*, and *Odoribacter* in the T group vs. the C group (Figure 6A). Figure 6B shows TD group had a greater genus level abundance of *Lactobacillus* and *Odoribacter* than that in the T group. 

## 4. Discussion

This study affords a new insight into the mechanisms behind perinatal TCDD-induced hypertension with specific emphasis on metabolites derived from gut microbes. Our study also highlights that maternal DMB therapy can be considered as a therapeutic intervention to prevent TCDD-induced programmed hypertension, which is a postbiotics-based approach to mediate gut microbiota-derived metabolites. 

Consistent with the result from prior work [6,7,8], the current study indicated perinatal TCDD exposure induced hypertension in 12-week-old male offspring. We found that not only TCDD exposure but also DMB treatment causes a decrease of body weight. Our data are in agreement with a previous study showing that in utero TCDD exposure reduced body weight in adult offspring [25]. However, whether the effect of DMB on offspring’s body weight is beneficial or harmful awaits further evaluation.

In the current study, perinatal TCDD exposure causes the rise of offspring’s BP coinciding with dysbiotic gut microbiota and impaired SCFAs production and receptor expression. In support of previous research indicating that gut microbiota dysbiosis contributes to hypertension [10,11,26], TCDD-induced hypertensive offspring displayed several microbial signatures such as reduced α-diversity, increased the F/B ratio, and a lesser abundance of beneficial microbes *Ruminococcus*, *Roseburia,* and *Odoribacter* [26,27]. On the other hand, maternal DMB therapy protects progeny against hypertension programmed by TCDD, which is related to alterations of gut microbiota composition, mediation of TMA-TMAO metabolic pathway, regulation of SCFA and their receptors, and restoration of the RAS and AHR signaling pathway.

Our data showed that major beneficial effects of DMB are relevant to alterations of gut microbiota and its metabolites. Maternal DMB therapy increased abundance of genera *Lactobacillus* and *Akkermansia*, both are known as beneficial gut microbes [26,28]. Despite an association between high F/B ratio and hypertension was reported [26], our study failed to identify a reduction of F/B ratio related to BP-lowering in the TD group. 

In this study, perinatal TCDD exposure had negligible effect on TMAO. Our finding suggests that the TMA-TMAO pathway is not a major determinant of hypertension in this model. Interestingly, early-life DMB treatment had long-term programming effect on TMA-related metabolites in adult male offspring. Our data demonstrated that DMB increased plasma TMA level, decreased TMAO-to-TMA ratio, and had no influence on TMAO level in the D and TD group. Considering TMAO levels are influenced by TMA formation and its degradation as well as secretion, the ratio between TMAO and TMA has been considered as a more relevant marker for cardiometabolic health rather than just TMAO levels [29]. However, the association between the TMAO-to-TMA ratio and hypertension remains unclear. Our data indicates DMB caused a decrease of TMAO-to-TMA ratio, suggesting programming effects of DMB are possibly relevant to increase TMA formation or decreased TMA-to-TMAO conversion. Of note, DMB is supposed to reduce TMA production in pregnant rats and, therefore, its increasing (but not decreasing) TMA on adult offspring seems a compensatory programming effect. In accordance with the alteration in TMA-TMAO pathway, alterations of the gut microbial community were observed. Prior studies reported that *Proteobacteria* is the major phylum capable of producing TMA from choline [14,30], which support the notion that DMB therapy augments *Proteobacteria* to increase TMA production.

We also examined other metabolites derived from gut microbiota, that is, SCFAs which can regulate BP through their receptors [13,23]. We found not only TCDD exposure but also DMB treatment caused increases of plasma acetic acid, which was augmented by combined TCDD + DMB exposure. Although our findings conflicting with prior studies reports that decreases of SCFAs may contribute to the pathogenesis of hypertension [9], the mechanism underlying TCDD-induced hypertension seems related to downregulation of SCFA receptor GPR43 and GPR109A. Generally speaking, SCFAs can increase BPs by stimulating GPR41 and Olfr78 to increase sympathetic nerve activity and increase renin secretion, respectively. Conversely, the elevation of BP can be counteracted through GPR43 and GPR109A to induce vasodilation [23]. The protective effect of DMB against hypertension is possibly due to it increases acetic acid and restores the decreased expression of GPR43 and GPR109A, in favor of vasodilatation.

Another positive effect of DMB therapy could be that it antagonizes AHR-mediated gene transcription. Prior research suggests AHR target genes might be involved in TCDD-induced hypertension [24]. Our results showed DMB protected hypertension coinciding with the restoration of TCDD-induced increased CYP1A1 expression. In view of that activation of the AHR/CYP1A1 axis can induce vasoconstriction [24], whether DMB antagonizes AHR/CYP1A1 to protect offspring against TCDD-induced hypertension deserves further clarification. Moreover, protective mechanism of DMB against TCDD-induced hypertension might be linked to restoration of the RAS balance, at least in part. The role of the activation of RAS in contributing to hypertension is well known. Our data showed that DMB protected hypertension is associated with the restoration of expression of AGT and ACE induced by TCDD. 

Overall, the present study has some limitations. First, the anti-hypertensive effects of DMB therapy might be attributed to other organs that regulate BP. Additional research is required to evaluate the programming effects of DMB on other BP-controlled organs. Another limitation is that we did not analyze TMA-related metabolites in dams because we are interested in DMB’s long-term effects on offspring instead of acute effects on mother rats. Third, we analyzed gut microbiota in adult offspring at the time hypertension appearing, but not in dams. Therefore, the dysbiosis characteristics of gut microbiota observed at 12-week-old offspring might be a consequence of programmed hypertension. Whether TCDD and DBM exposure to mothers might alter the gut microbiota in both dams and offspring, and whether maternal gut microbiota is connected with offspring outcome, both require further evaluation. Fourth, we observed that DMB and TCDD decreased body weight. However, their programming effect on the BW of offspring remains unclear. Therefore, further studies are needed to determine whether their BW-lowering effect is beneficial or harmful. Furthermore, we only measure plasma creatinine level, a thorough examination of renal outcome (e.g., proteinuria, blood urea nitrogen, and renal pathology) is worthy further study to determine whether adult offspring exposed to TCDD develops early stage of chronic kidney disease. Lastly, we did not examine other timing or dosing of DMB, whether these alterations produce similar or different programming effects on TCDD-induced hypertension await further investigation.

## 5. Conclusions

In summary, there are several key mechanisms behind the protective actions of DMB on the adult offspring perinatally exposed to TCDD, including reshaping gut microbiome; mediating SCFAs via increasing acetic acid, restoring SCFA receptor GPR43 and GPR109A expression; antagonizing AHR-mediated CYP1A1 expression; and balancing the RAS in favor of vasodilatation. Most importantly, our findings illuminate the therapeutic potential of postbiotics targeting on microbial metabolites for the prevention of hypertension of developmental origins. 

## Figures and Tables

**Table 1:** List of primer sequences used for qPCR analysis.

Gene	Forward	Reverse
GPR41	TCTTCACCACCGTCTATCTCAC	CACAAGTCCTGCCACCCTC
GPR43	CTGCCTGGGATCGTCTGTG	CATACCCTCGGCCTTCTGG
GPR109A	CGGTGGTCTACTATTTCTCC	CCCCTGGAATACTTCTGATT
Olfr78	GAGGAAGCTCACTTTTGGTTTGG	CAGCTTCAATGTCCTTGTCACAG
Renin	AACATTACCAGGGCAACTTTCACT	ACCCCCTTCATGGTGATCTG
AGT	GCCCAGGTCGCGATGAT	TGTACAAGATGCTGAGTGAGGCAA
ACE	CACCGGCAAGGTCTGCTT	CTTGGCATAGTTTCGTGAGGAA
ACE2	ACCCTTCTTACATCAGCCCTACTG	TGTCCAAAACCTACCCCACATAT
AT1R	GCTGGGCAACGAGTTTGTCT	CAGTCCTTCAGCTGGATCTTCA
AT2R	CAATCTGGCTGTGGCTGACTT	TGCACATCACAGGTCCAAAGA
AHR	GTCCTCAGCAGGAACGAAAG	CCAGGGAAGTCCAACTGTGT
AHRR	CAGCAACATGGCTTCTTTCA	TGAAGCACTGCATTCCAGAC
CYP1A1	GCACTCTGGACAAACACCTG	ATATCCACCTTCTCGCCTGG
ARNT	GTCTCCCTCCCAGATGATGA	GCTGGTAGCCAACAGTAGCC
TIPARP	GTTGAGGGCCAATTACCAGA	GCTCCTGGCACATAATCCAT
R18S	GCCGCGGTAATTCCAGCTCCA	CCCGCCCGCTCCCAAGATC

Oflr78 = olfactory receptor 78; GPR41 = G protein-coupled receptor 41; GPR43 = G protein-coupled receptor 43; GPR109A = G protein-coupled receptor 109A; AGT = angiotensinogen; ACE = angiotensin converting enzyme; ACE2 = angiotensin converting enzyme-2; AT1R = angiotensin II type 1 receptor; AT2R = angiotensin II type 2 receptor; AHR = aryl hydrocarbon receptor, ARNT = Aryl hydrocarbon receptor nuclear translocator, AHRR = Aryl hydrocarbon receptor repressor, TIPARP = TCDD-inducible poly-ADP-ribose polymerase, CYP1A1 = cytochrome P450 CYP 1A1, R18S = 18S ribosomal RNA.

**Table 2:** Weight, renal function, and blood pressures.

Groups	C	D	T	TD
Body weight (BW) (g)	360 ± 13	311 ± 8 *	309 ± 7 *	286 ± 14 *
Left kidney weight (g)	1.57 ± 0.08	1.2 ± 0.07 *	1.51 ± 0.07	1.37 ± 0.08
Left kidney weight/100g BW	0.44 ± 0.01	0.49 ± 0.01	0.49 ± 0.02	0.48 ± 0.01
Systolic BP (mmHg)	133 ± 1	133 ± 1	143 ± 1 *	135 ± 1 ^#^
Diastolic BP (mmHg)	90 ± 2	86 ± 2	91 ± 2	86 ± 2 ^#^
MAP (mmHg)	104 ± 1	102 ± 2	108 ± 1 *	103 ± 1
Creatinine (μM)	16.4 ± 0.5	17.4 ± 0.9	15 ± 0.7	16.2 ± 0.6

*N* = 8 per group; * *p* < 0.05 vs. C; ^#^ *p* < 0.05 vs. T. BP = blood pressure. MAP = mean arterial pressure.

**Table 3:** Plasma trimethylamine (TMA), trimethylamine-N-oxide (TMAO), and dimethylamine (DMA) levels.

Groups	C	D	T	TD
DMA (ng/mL)	117 ± 10	132 ± 11	118 ± 9	146 ± 15
TMA (ng/mL)	564 ± 23	810 ± 29 *	593 ± 28	844 ± 57 *
TMAO (ng/mL)	440 ± 23	444 ± 20	387 ± 20	429 ± 35
TMAO-to-TMA ratio	0.78 ± 0.02	0.55 ± 0.03 *	0.66 ± 0.04	0.53 ± 0.06 *
DMA-to-TMAO ratio	0.27 ± 0.02	0.3 ± 0.03	0.31 ± 0.03	0.35 ± 0.03

*N* = 8 per group; * *p* < 0.05 vs. C.

**Table 4:** Plasma concentrations of short chain fatty acids (SCFAs).

Groups	C	D	T	TD
Acetic acid (ng/mL)	169.9 ± 10	232.3 ± 6 *	214.7 ± 10.1 *	288.5 ± 16.4 *^,#^
Propionic acid (ng/mL)	1.43 ± 0.06	1.4 ± 0.16	1.24 ± 0.23	1.36 ± 0.09
Butyric acid (ng/mL)	1.44 ± 0.13	1.28 ± 0.05	1.38 ± 0.07	1.32 ± 0.08

*N* = 8 per group; * *p* < 0.05 vs. C; ^#^ *p* < 0.05 vs. T.

## Data Availability

Data will be available upon request.
